# Post-COVID-19 Action: Guarding Africa’s Crops against Viral Epidemics Requires Research Capacity Building That Unifies a Trio of Transdisciplinary Interventions

**DOI:** 10.3390/v12111276

**Published:** 2020-11-09

**Authors:** Francis O. Wamonje

**Affiliations:** International Centre of Insect Physiology and Ecology (*icipe*), Nairobi 30772-00100, Kenya; fwamonje@icipe.org; Tel.: +254-726-225272

**Keywords:** plant viruses, high-throughput sequencing, diagnostics, plant-virus-vector interactions

## Abstract

The COVID-19 pandemic has shown that understanding the genomics of a virus, diagnostics and breaking virus transmission is essential in managing viral pandemics. The same lessons can apply for plant viruses. There are plant viruses that have severely disrupted crop production in multiple countries, as recently seen with maize lethal necrosis disease in eastern and southern Africa. High-throughput sequencing (HTS) is needed to detect new viral threats. Equally important is building local capacity to develop the tools required for rapid diagnosis of plant viruses. Most plant viruses are insect-vectored, hence, biological insights on virus transmission are vital in modelling disease spread. Research in Africa in these three areas is in its infancy and disjointed. Despite intense interest, uptake of HTS by African researchers is hampered by infrastructural gaps. The use of whole-genome information to develop field-deployable diagnostics on the continent is virtually inexistent. There is fledgling research into plant-virus-vector interactions to inform modelling of viral transmission. The gains so far have been modest but encouraging, and therefore must be consolidated. For this, I propose the creation of a new Research Centre for Africa. This bold investment is needed to secure the future of Africa’s crops from insect-vectored viral diseases.

## 1. Preamble: COVID-19 Is a Global Wake-Up Call on Preparedness for Viral Pathogens

The ability to detect viral pathogens accurately and speedily has been thrust into global importance with the advent of the coronavirus SARS-CoV-2, the causative agent of COVID-19 disease [1]. High-throughput sequencing (HTS) (colloquially termed “deep sequencing”) has generated whole-genome information about SARS-CoV-2 useful in the development of diagnostic tests and informing public health decision-making [2]. Rapid detection tests have been proved a valuable and practical approach in conducting mass testing to generate epidemiological data that can be used in modelling of virus spread [3,4]. Unfortunately, most diagnostic platforms are not versatile or cost effective enough to scale testing throughput without substantial investment in equipment and skilled human resources [5,6]. Reducing transmission between humans has been a critical control measure, predicated on nonpharmaceutical interventions such as physical distancing and ventilation [7]. These interventions, instituted globally, have been painful to the world population but are deemed essential [8].

Similar challenges can affect agriculture. Pathogens and pests can leave most parts of the globe vulnerable [9,10]. A severe viral epidemic would, at the very least, severely undermine progress in achieving sustainable development goals 1, 2 and 3 (no poverty, zero hunger and good health and wellbeing, respectively). Therefore, the COVID-19 outbreak should serve as a wake-up call for governments worldwide. The most robust systems can be overwhelmed, and no country is immune to viral pandemics. The outbreak scenario is especially real for insect-vectored plant viruses, which can rapidly achieve transboundary spread. In Africa, insect-vectored viruses such as whitefly transmitted begomoviruses can spread across borders and cause widespread losses of essential staples, such as cassava (*Manihot esculenta*) [11]. Lessons from COVID-19 underscore the importance of a unified and long-term approach to prepare for “pandemic-like” events. For insect-vectored plant viruses, this can be predicated on a trio of transdisciplinary interventions: (1) using HTS to detect circulating viruses and discover new ones, (2) creation of versatile and scalable diagnostic capabilities, and (3) research into plant-virus-vector interactions to inform mathematical modelling of virus spread.

## 2. Current State of Africa’s Research in Virus Discovery, Diagnostics, and Plant-Virus-Vector Interactions

### 2.1. HTS-Driven Virus Discovery

In Africa, HTS has been used for the detection of viruses from crops such as common bean (*Phaseolus vulgaris*) [12,13,14,15,16], maize (*Zea mays*) [17,18,19], sweet potato (*Ipomoea batatas*) [20,21,22], papaya (*Carica papaya*) [23], pumpkin (*Cucurbita pepo*) [24,25], fluted pumpkin (*Telfairia occidentalis*) [26], cassava [27,28,29], yam (*Dioscorea* spp.) [30,31], cowpea (*Vigna unguiculate*) [32] and in Poaceae plants [33]. These forages into HTS have led to the detection of known viruses and the discovery of many novel virus species (Table 1). Infrastructure and access to HTS sequencing facilities remain limited. In East and Central Africa, the most active hub for plant HTS is the Kenya-based Biosciences eastern and central Africa hub (BecA-ILRI) at the International Livestock Research Institute (ILRI). ILRI is an international research organization under the Consultative Group in International Agriculture Research (CGIAR). Most of the time, deep sequencing is outsourced overseas or conducted through international partnerships and collaborations in Western-based sequencing facilities (Table 1). There is at least one privately run commercial HTS provider in Kenya, The African Genomics Centre and Consultancy, which is probably the only such facility in Sub-Saharan Africa outside of South Africa. “Third-generation” sequencers, such as the MinION from Oxford Nanopore, are now gaining a foothold. These portable sequencers have been used for the detection of cassava viruses in “field conditions” by researchers in East Africa [34]. MinION sequencers are relatively affordable (about US $1000 each), and this could improve and increase access to HTS sequencing.

Commensurate to HTS, bioinformatics training for plant sciences is important for African-based researchers. Most bioinformatics training initiatives including short courses and virtual learning platforms have targeted human health interventions [35,36,37]. Similarly, publicly available online analysis platforms for plant viruses similar to VirFind [38] need to be established with Africa to ease the analysis of HTS data. These bioinformatics analysis servers for plant viruses are usually hosted within a university or research institution where considerable HTS sequencing is done.

### 2.2. Plant Virus Diagnostics

Most laboratory plant virus diagnostics in Africa have been reliant on serology and notably Enzyme-Linked Immunosorbent Assay (ELISA) [44]. ELISA enjoys universal usage in most testing laboratories due to its repeatability and reproducibility. However, it has its weaknesses, including the dependency on high-quality antisera, whose generation requires specialised laboratories and challenges in using antisera to resolve virus identities for closely related strains [44]. ELISA is also not versatile enough for large-scale field deployment. Other serological tests such as portable diagnostic strips tests (immunostrips), when available, offer greater utility value in monitoring disease incidence (see later section on Maize Lethal Necrosis).

Most immunostrips are imported from the United States and Europe. There is almost no capacity to produce these strip tests for plant viruses anywhere in Africa, although their manufacture is not complex and some can literally be ‘’ink-jet’’ printed [45]. The COVID-19 pandemic has revealed that supply chain disruptions and a sudden surge in demand for reagents can severely affect a country’s ability to test and manage a viral disease. It is mission-critical for Africa to have the homegrown capacity to develop and manufacture diagnostic kits and, especially, immunostrips.

Virus genome information has been used by Africa’s researchers to create laboratory-based molecular diagnostics utilizing polymerase chain reaction (PCR) and reverse-transcription-PCR (RT-PCR) for staples, such as cassava, sweet potato and rice (*Oryza sativa*), and valuable commercial crops, such as tomato (*Solanum lycopersicum*) [46,47,48,49,50,51]. With HTS generated virus genome information, more can be achieved. For most HTS projects, molecular tests, usually by PCR and RT-PCR are developed to confirm the presence of a detected virus. PCR and RT-PCR assays provide a good starting point in utilization of sequence information from HTS to generate primer sequences, optimize thermocycling conditions and validate positive controls.

However, PCR-based techniques, cannot be rapidly scaled in the event of an epidemic without exacting high cost and infrastructural demands. International organizations, such as ILRI, the Nigeria-headquartered International Institute for Tropical Agriculture and the International Centre of Insect Ecology and Physiology (*icipe*) in Nairobi, have historically had the best facilities to support virus molecular diagnostics work. This is changing, notably through two initiatives in West Africa. First is the Laboratoire Mixte International PathoBios, LMI PathosBios (www.pathobios.com), in Burkina Faso. Created in 2013, as a partnership to universities of Burkina Faso with support from France and Belgium, LMI PathoBios, runs two research platforms (with molecular laboratories) on two sites in Burkina Faso. These research platforms make it possible to carry out molecular diagnostics, microbiology, and plant experimentation for researchers in Burkina Faso and other countries in the subregion. Another is the West Africa Virus Epidemiology, WAVE (https://wave-center.org/), headquartered in Cote d’Ivoire with membership from the nations of Benin, Burkina Faso, Democratic Republic of Congo, Ghana, Nigeria and Togo. To improve early detection and diagnosis of plant viruses, laboratories have been built or refurbished and equipped in all institutions hosting the WAVE program in all the member countries. In Côte d’Ivoire, diagnostics facilities comprising state-of-the-art laboratories for molecular virology were opened in 2016. These timely and impactful capacity investments are increasing the capacity of these west African nations to detect and diagnose plant viruses.

### 2.3. Plant-Virus-Vector Interactions Research

In-depth studies on how insects vector plant viruses are a rarity in Africa. Some of these, led by the United Kingdom (UK)-based researchers have enabled a deeper understanding of virus vectoring by aphids and whiteflies and detailed mathematical modelling [52,53,54]. More recently, there has been an initiative to form an Africa-wide network to encourage their collaboration with the UK-based researchers working on insect-vectored plant viruses. The UK funded and led “Community Network for African Vector-Borne Plant Viruses” (CONNECTED) network has reinvigorated plant-virus-vector interactions research in Africa (see www.connectedvirus.net). Through this initiative, pump-priming grants supporting 20 research projects, in 14 countries, involving 11 different crops, and collaborations of 55 researchers in 34 institutions are in various stages of execution. Through CONNECTED, training, mentorship and networking opportunities have grown. The end of its funding cycle should not lead to the demise of the gains made. Increased use of such networks increases visibility of African researchers, enhances knowledge sharing and promotes “south–south” research connections.

### 2.4. Learning from Past Application of Transdisciplinary Interventions in Addressing Plant Virus Challenges

The key question from this synthesis is: how can we consolidate and enhance the gains in HTS, improve diagnostics and sustain studies into insect vectoring of plant viruses for the benefit of Africa’s crops? There are currently no research centres designed to host these three research areas as their core functions anywhere on the African continent. Bold proposals and investments are needed to consolidate these three research areas into long-term action so that crops protection in Africa can be proactive rather than reactive. In subsequent sections, I will use two essential staples, i.e., maize and common bean as case studies to demonstrate the value of HTS, diagnostics and studies into virus vectoring by insects.

#### 2.4.1. Case Study 1. Maize Lethal Necrosis Disease

In Sub-Saharan Africa, maize is a crucial staple to more than 300 million people. It accounts for 73% of the total starch demand in Eastern and Southern Africa and 64% in Western and Central Africa [55]. Recently, maize production in parts of Eastern and Southern Africa was severely affected by a viral disease called Maize Lethal Necrosis (MLN). This aggressive disease results from coinfection of maize with Maize chlorotic mottle virus (MCMV) (*Tombusviridae*) and members of the *Potyviridae* family: Sugarcane mosaic virus (SCMV), Maize dwarf mosaic virus (MDMV), Johnsongrass mosaic virus (JGMV) or the Tritimovirus, Wheat streak mosaic virus [56].

The discovery of MLN in Kenya in 2011 and its rapid spread to Rwanda, Democratic Republic of Congo, Tanzania, Uganda, South Sudan and Ethiopia was a reminder of how acute food crises can be caused by viruses [56,57]. In Kenya, yield losses in 2012 resulted in an estimated loss of 126,000 metric tons valued at 52 million USD [57]. In the following year (2013), half the maize crop in Western Kenya was lost, and cumulatively, countrywide losses were modelled at about 500,000 metric tons or about 22% of total maize production [56]. The overall economic impact on smallholder farmers in the Eastern Africa region encompassing Kenya, Uganda, Tanzania, Rwanda and Ethiopia was estimated to be between 291 and 339 million USD [56].

From early on, HTS was used to decipher the diversity of the circulating viral components of MLN. From the genome information, RT-PCR diagnostics were developed [58]. Whole-genome information revealed that getting reliable tests for SCMV would be tricky due to high genetic diversity and virus recombination [41]. Therefore, the most reliable tests for MLN are anchored on detection of MCMV, which is risky. A mutation in MCMV can render current detection tools obsolete.

In the MLN management strategy, portable diagnostic strips tests for MCMV were useful in collecting data on the spread of the virus across different countries. In addition, significant resources were used to identify maize cultivars that were resistant or tolerant to the viral components of MLN [59]. MCMV diagnostic strip tests were extensively used in commercial seed production fields with closest attention to virus-tolerant maize cultivars that may be asymptomatic while harbouring MLN components [59]. Rapid testing enabled seed companies to reduce the likelihood of introducing new sources of infection into the field via seed. The availability of these rapid tests allowed independent monitoring by other agencies for compliance to agree MLN management guidelines [59].

However, in the absence of studies into the vectoring of MLN viruses by insects, the current state of MLN in the Eastern Africa region could end up being akin to dodging a bullet. MCMV is vectored by chrysomelid beetles and thrips (*Frankliniella*), while the potyviral components are vectored by a variety of aphids such as *Rhopalosiphum maidis*, *R. padi*, *Myzus persicae* and *Schizaphis graminum* [56]. Since thrips and aphids differ considerably in life histories, dispersal abilities, survival on different hosts and virus transmission characteristics, experiments giving this biological information are important for better modelling of their transmission of these viruses [60].

#### 2.4.2. Case Study 2. Common Bean: Recombinant Viruses and New Viral Threats

Common bean is a principal source of dietary protein for millions of people in Africa. In some areas of Eastern Africa, such as Rwanda, Burundi, Uganda and Kenya, consumption by individuals can be as high as 40–66 kg per year [61]. In Africa, Bean common mosaic virus (BCMV), Bean common mosaic necrosis virus (BCMNV) and Cucumber mosaic virus (CMV) are the most important threats to bean production [12,62]. Aphids transmit these three viruses. Controlling BCMV and BCMNV has been mainly through breeding for resistance by pyramiding, in various combinations, the dominant *I* gene and a panel of recessive genes. There are now recombinant isolates of BCMV that can overcome recessive resistance conferred by *bc-3*, *bc-1* and *bc-2* genes, induce temperature-independent necrosis and cause unusually severe bean pathology [62]. These findings hold serious negative implications for the management of these bean-infecting viruses.

There has been HTS-driven discovery of “Asiatic” strains of CMV from bean plants in Kenya [12]. The researchers identified this CMV strain as a reassortant virus comprising an RNA 3 belonging to CMV subgroup IB (which are predominantly Asian in their distribution) and subgroup IA RNAs 1 and 2, also likely of Asian origin. The appearance of exotic CMV strains may represent a serious threat to Africa’s crops. CMV has a remarkably wide host range, including essential African staples, some of which are intercropped with beans such as maize [63], banana [64] and sweet potato [65].

Other new or understated viral threats have discovered through deep sequencing. Researchers in Tanzania and Zambia detected Southern bean mosaic virus (SBMV), a Sobemovirus, that causes up to 50% yield loss in some bean varieties [13,39,66]. This Chrysomelid beetle-vectored virus has previously caused losses to bean farmers in Morocco [67]. The Tanzanian researchers also detected Tomato leaf curl Uganda virus (ToLCUV) [13]. This ToLCUV discovery is of interest since it shows that this virus could have a broad host range, including common bean. ToLCUV, first reported in 2003 following the observation of leaf curl symptoms in tomato in Uganda is a recombinant and possibly a novel begomovirus [68].

It is now widely accepted that viruses can affect insect choice of host plants and feeding behaviour to influence their onward transmission [69]. Advances have been made in understanding insect vectoring of bean viruses by aphids. Recently, researchers have used electrical penetration graph (EPG) technique to study virus-mediated changes to the feeding behaviour of aphids (*Aphis fabae* and *Myzus persicae*) on common bean [53]. They reported that though virus-infected plants engendered different feeding behaviours, the overall effects would promote the transmission of the viruses [53]. Mathematical modelling of aphid transmission of viruses, based on aphid feeding, fecundity and other biological insights, showed that virus modifications influence epidemics by altering vector distribution, density and even vector form [52]. The insights from these models are essential for understanding how pathogens, in general, propagate through natural plant communities and crops and have practical use in devising novel strategies to reduce losses by disrupting the transmission of viruses.

## 3. What a New Research Centre Could Do for Africa

Both cases studies reveal research gaps; vector studies, in the case of maize, and diagnostics (especially for newly discovered viruses) in common bean. Investing in a New Research Centre for Africa, anchored around three research pillars, i.e., HTS, diagnostics and plant-virus-vector interactions, is key to guiding and hosting long-term research effort to find new ways to protect Africa’s crops. The activities within the three pillars should complement each other to provide a comprehensive suite of solutions for crop protection.

### 3.1. High-Throughput Sequencing: A Lynchpin in Preparedness for Viral Diseases

This research pillar aims to provide information that will ultimately forewarn and forearm farmers, policymakers and researchers on new viruses and changes in the genomics of known viruses. Advanced molecular laboratories with HTS technologies and capability are required for virus discovery through metagenomic approaches. Appropriately planned and targeted HTS activities will lead to (1) the discovery of novel viruses and detection of known viruses that could be spreading undetected, (2) mapping the species diversity of the discovered viruses and their presence in different hosts and trophic levels within cropping ecosystems and (3) providing whole-genome sequence information of the detected viruses to inform genomics-driven creation of diagnostic tools.

### 3.2. Diagnostics: Enabling Crowdsourcing of Virus Incidence

This research pillar will enable and increase the use of diagnostics as a primary tool of viral disease surveillance and management. Rapid diagnostics will empower farmers to test for diseases on the farm as soon as they identify “disease-like” symptoms. This way, virus incidence data can be crowdsourced. Essential activities in this research pillar should include (1) development, and manufacture, of rapid diagnostic tests and kits, (2) maintenance of a growing repository of positive controls for viral pathogens accessible to researchers from all over Africa, (3) generation of PCR primers, probes, conventional PCR, RT-PCR and quantitative PCR tests and (4) research into other novel diagnostic capabilities utilizing nanotechnology.

### 3.3. Plant-Virus-Vector Interactions Research for Modelling and Disruption of Virus Transmission

The goal of this research pillar is to generate biological information that can be used in mathematical modelling of virus transmission by insects. This information is essential in moving farming toward sustainable production systems by preventing or reducing insect vectoring of viruses. Continual experimentation in ways of disrupting vectoring that utilize chemical ecology, barrier plants, insect host ranges and biocontrol measures is required. Facilities such as laboratories, insectaries, climate-controlled plant growth chambers, glasshouses, large insect arenas and greenhouses are needed. These facilities will enable (1) year-round growth of critical food crops and model plants (such as Arabidopsis (*Arabidopsis thaliana*) and Tobacco (*Nicotiana* sp.)) for experimentation, (2) maintenance of insect stocks, (3) experimental simulation of different biotic and abiotic conditions on location and (4) mathematical modelling. These capabilities, collectively, can be used to understand the threats posed by different insects with respect to virus transmission and crucially whether the interventions we devise will make things better or worse.

## 4. Practical Steps Forward

It is timely that a New Research Centre for Africa is established. To emulate the momentum in West Africa, in improving both human capacity and infrastructure (by LMI PathoBios and WAVE), a first centre could strategically be in Kenya, East Africa. Kenya enjoys good infrastructure, a stable economic and political climate and excellent communication connectivity for IT and travel. In addition, the presence of two CGIAR centres has made Kenya a hub for international research. The new centre would be used to perfect a phased approach in assembling a core of researchers and acquisitions of equipment and technologies needed to build momentum in all three research areas. The goal would be to get from concept to a working model Centre with ongoing research along with the three thematic areas of virus discovery, diagnostics and plant-virus-vector interactions within 5 years. This “working model” can then be replicated in other African countries such that a network of fully-fledged research campuses with space, infrastructure, researchers, and resources become a reality.

## Figures and Tables

**Table 1:** Recent use of high-throughput sequencing (HTS) for virus discovery in some of Africa’s food crops.

Crop Ecosystem	Country Origin of Samples	Country Where Sequencing Was Done	Sequencing Provider	Key Viruses Detected	Reference(s)
Common Bean	Kenya	Kenya	BecA-ILRI	Bean common mosaic necrosis virus (BCMNV), Cucumber mosaic virus (CMV), Phaseolus vulgaris endornavirus 1 (PvEV-1), Phaseolus vulgaris endornavirus 2 (PvEV-2)	[12]
	Kenya	Australia/Korea	Macrogen	BCMNV, Cowpea aphid-borne mosaic virus (CABMV), Pelargonium vein banding virus (PVBV), Dracaena mottle virus (DrMV), Lucky bamboo bacilliform virus	[14,15]
	Tanzania	Switzerland	Fasteris	Bean common mosaic virus (BCMV), BCMNV, Southern bean mosaic virus (SBMV), Tomato leaf curl Uganda virus (ToLCUV), PvEV-1, PvEV-2	[13]
	Zambia	South Africa	Inqaba	SBMV	[39]
	Kenya	Kenya	BecA-ILRI	Aphid lethal paralysis virus, new virus tentatively named “Big Sioux River-virus-like dicistrovirus”	[16]
Maize	Kenya	Kenya	BecA-ILRI	MCMV, SCMV, Maize streak virus (MSV) and Maize yellow dwarf virus-RMV (MYDV-RMV), Hubei Poty-like virus 1, Barley virus G, Scallion mosaic virus and Johnson grass mosaic virus (JGMV)	[17]
	Tanzania	Kenya	BecA-ILRI	MCMV, SCMV, MYDV-RMV, Maize dwarf mosaic virus (MDMV), Sorghum mosaic virus, Barley yellow dwarf virus	[18]
	Kenya Uganda Rwanda Tanzania			JGMV, MDMV, MCMV	[19]
	Kenya	China	Beijing Genomics Institute (BGI)	MCMV and SCMV	[40,41]
Cassava	Tanzania	Kenya	BecA-ILRI	Cassava brown streak virus (CBSV) and Ugandan cassava brown streak virus (UCBSV)	[27]
	Kenya	Kenya	BecA-ILRI	Cassava mosaic “geminiviruses”	[29]
	Kenya, Uganda and Tanzania	Kenya	Field sequencing using a portable MinION	Cassava mosaic begomoviruses	[34]
	Uganda	Australia	Australian Genome Research Facility (AGRF)	CBSV and UCBSV	[28]
Yam	Ghana and Nigeria	United Kingdom	Unknown	Yam mosaic virus, New virus tentatively named “Yam virus Y,” New badnaviruses-like viruses tentatively named “Dioscorea bacilliform RT virus, isolate DBRTV3-[2RT]” and “Dioscorea bacilliform RT virus, isolate DBRTV3-[3RT]”	[30,31]
Sweet potato	Kenya, Rwanda, Tanzania and Uganda	Kenya	BecA-ILRI	Sweet potato feathery mottle virus (SPFMV)	[20]
	Tanzania	Finland	Haartman Institute, University of Helsinki	SPFMV, Sweet potato virus 2, Sweet potato latent virus, Sweet potato mild mottle virus, Sweet potato virus G, Sweet potato badnavirus A, Sweet potato badnavirus B, Sweet potato badnavirus C, Sweet potato chlorotic fleck virus, Sweet potato chlorotic stunt virus Sweet potato begomovirus, Sweet potato caulimo-like virus, Sweet potato geminivirus, Sweet potato leaf curl Spain virus, Sweet potato mosaic associated virus, Sweet potato leaf curl Uganda virus, Sweet potato golden vein associated virus, Sweet potato leaf curl virus	[42]
	South Africa	South Africa	Unknown	SPFMV, Sweet potato virus G (SPVG), Sweet potato virus C (SPVC), Sweet potato chlorotic stunt virus (SPCSV), Sweet potato leaf curl Sao Paulo virus (SPLCSPV), Sweet potato caulimo-like virus (SPCV) and Sweet potato mosaic virus (SPMaV), Sweet potato badnavirus A and Sweet potato badnavirus B	[21,22]
	Kenya	Australia/Korea	Macrogen	SPFMV, SPVC, SPCSV, Sweet potato chlorotic fleck virus (SPCFV)	[43]
Papaya	Kenya	Kenya	BecA-ILRI	Moroccan watermelon mosaic virus (MWMV) Cowpea mild mottle virus (CpMMV) Two putative Carlaviruses tentatively named “Papaya mottle-associated virus (PaMV)” and “Papaya mild mottle-associated virus (PaMMV)”	[23]
Pumpkin	Kenya	Kenya	BecA-ILRI	MWMV A putative novel member of the genus Polerovirus tentatively named “pumpkin polerovirus” (PuPV)	[24]
	South Africa	South Africa	Agricultural Research Council Biotechnology Platform (ARC-BTP)	New polerovirus tentatively named “Pepo aphid-borne yellows virus (PABYV)”	[25]
Fluted pumpkin	Cameroon	United States of America	Unknown	New begomovirus tentatively named “Telfairia mosaic virus (TelMV)”	[26]
Cowpea	Burkina Faso	France	Unknown	CABMV, BCMV, Cowpea mottle virus (CPMoV), Southern cowpea mosaic virus (SCPMV), New Cowpea-associated tymovirus-like viruses, Cowpea-associated tombusvirids and Cowpea-associated poleroviruses tentatively named “Cowpea polerovirus 1, Cowpea polerovirus 2, Cowpea tombusvirid 1, Cowpea tombusvirid 2, Cowpea-associated mycotymovirid 1”	[32]
Poaceae plants	Reunion Island	United States of America	GENEWIZ	Mastrevirus species (African streak viruses) and three novel Mastrevirus species tentatively named “Eleusina indica-associated virus, Sorghum arundinaceum-associated virus, Melinis repens-associated virus”	[33]
